# Supplementary figures and images for: Seedling Responses to Organically-Derived Plant Growth Promoters: An Effects-Based Approach
Source: Plants (Basel). 2021 Mar 30;10(4):660. doi: 10.3390/plants10040660 (PMC8066269; doi:10.3390/plants10040660)

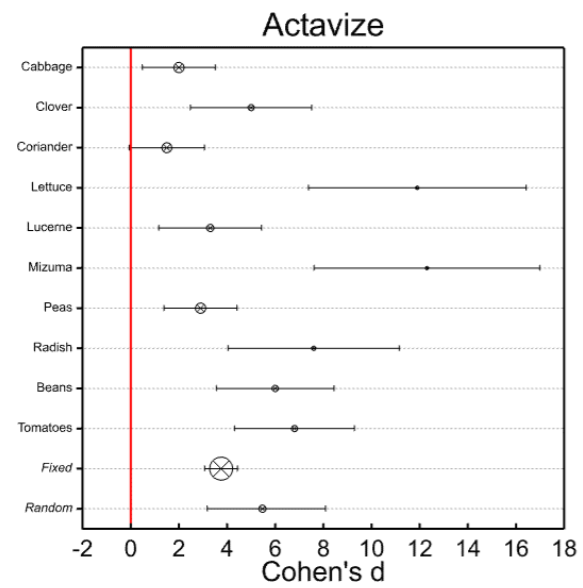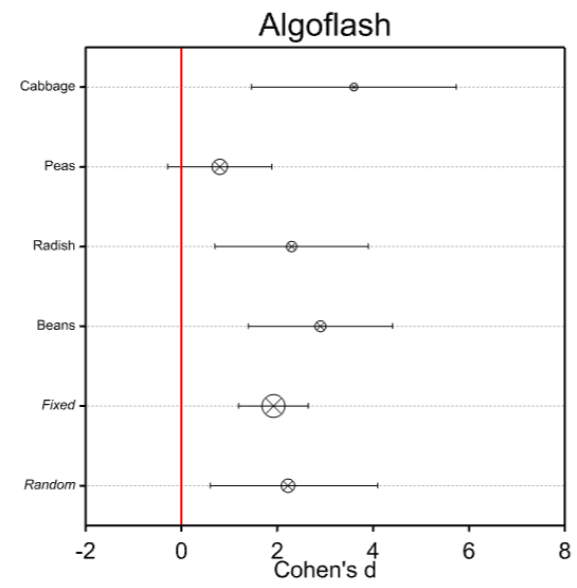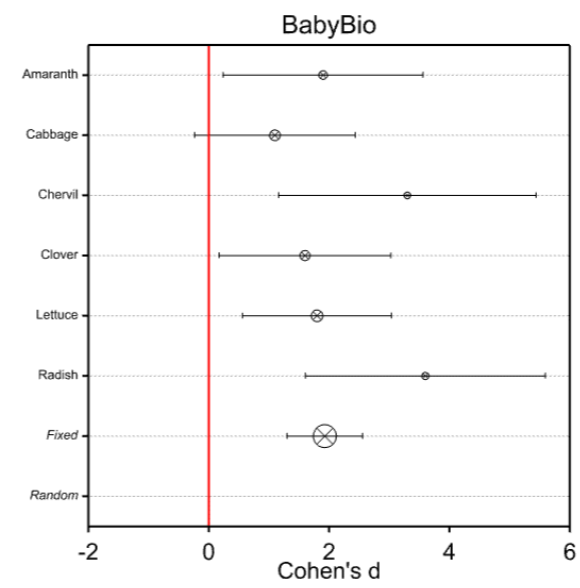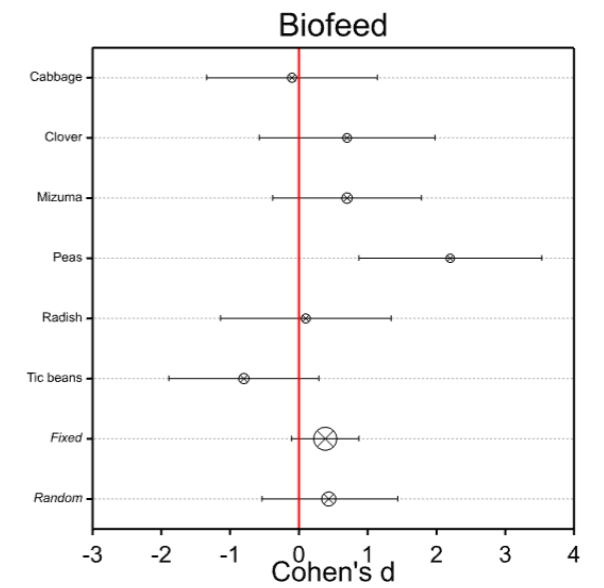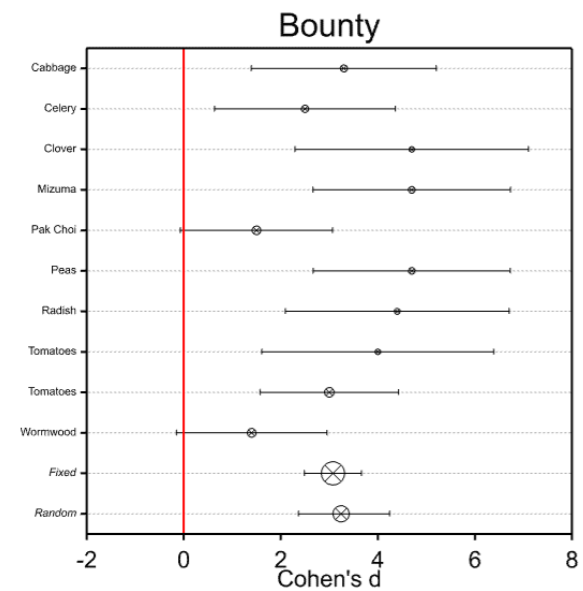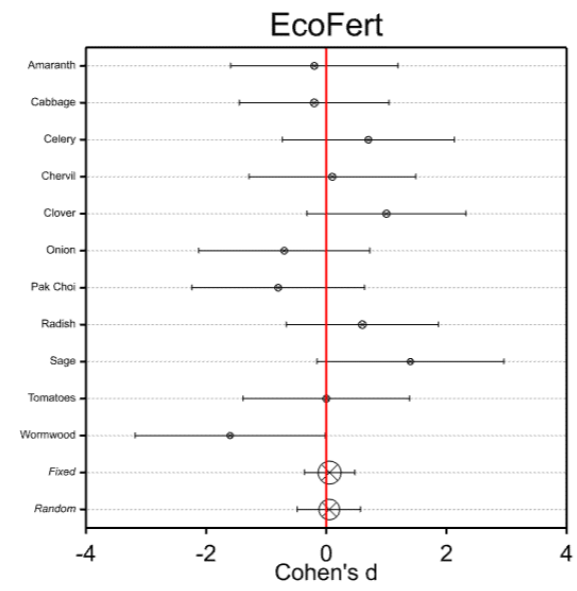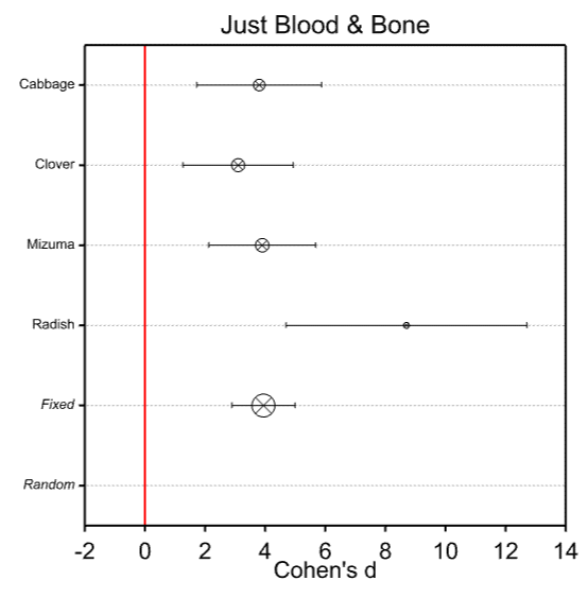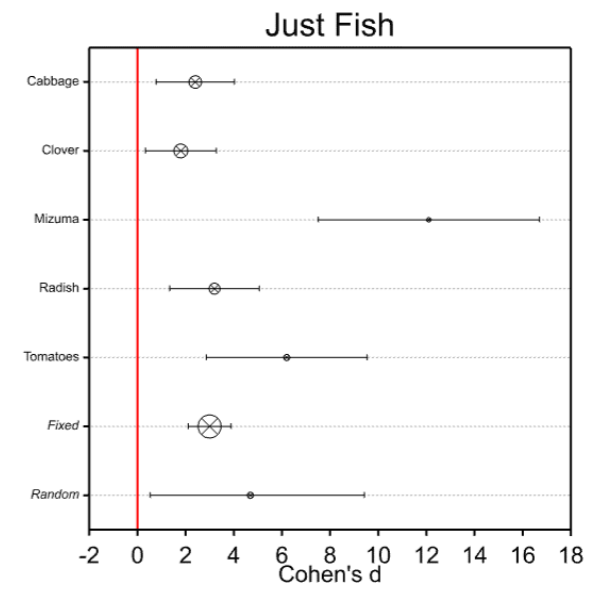

Nourish

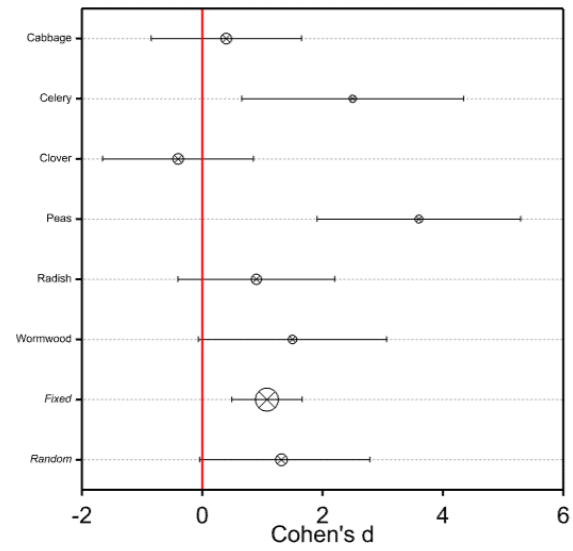

Phostrogen

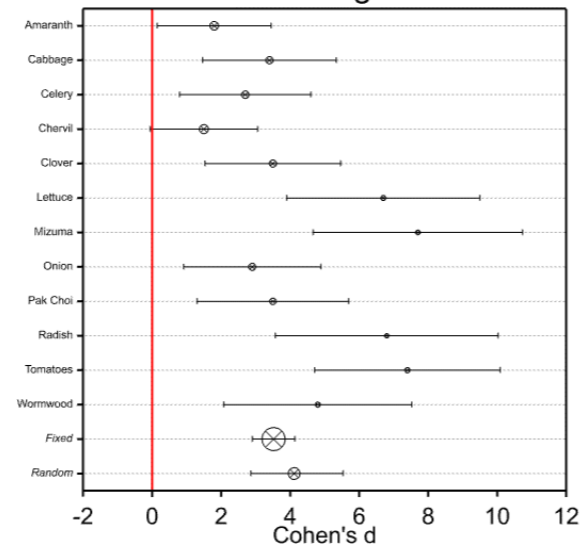

Seasol

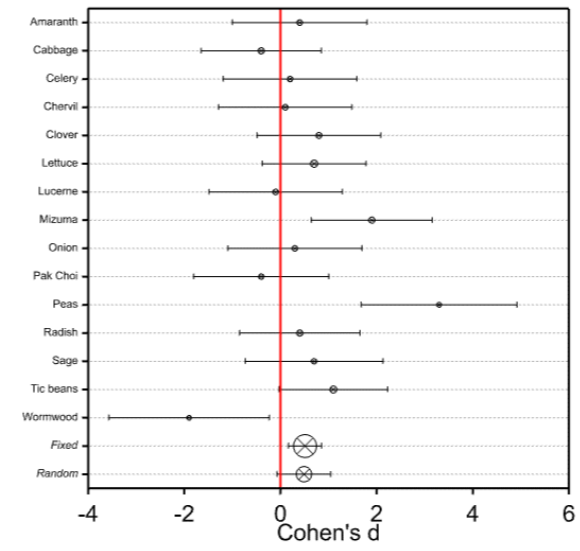

Synerlogic

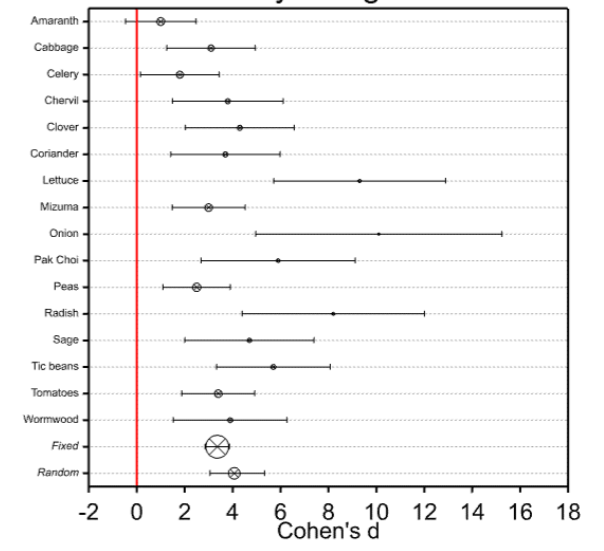

Vertesea

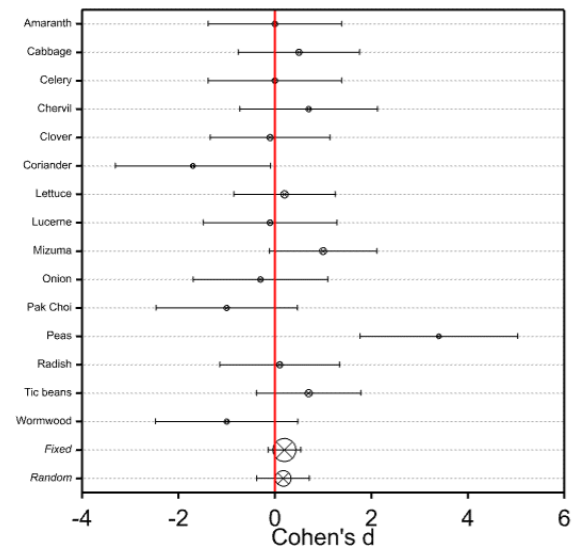

Wondergrow

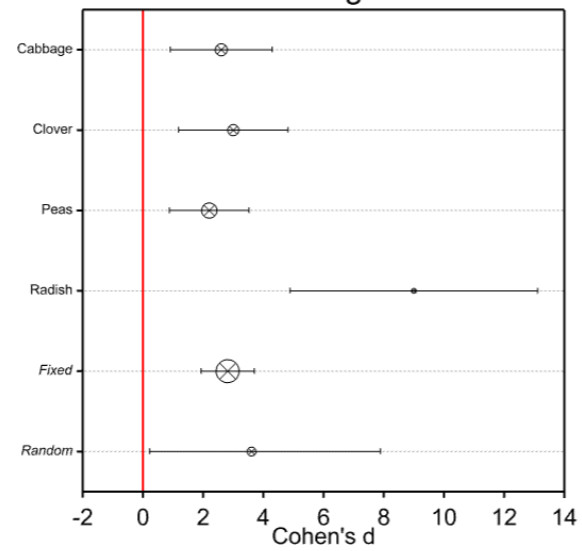

Yates Fish

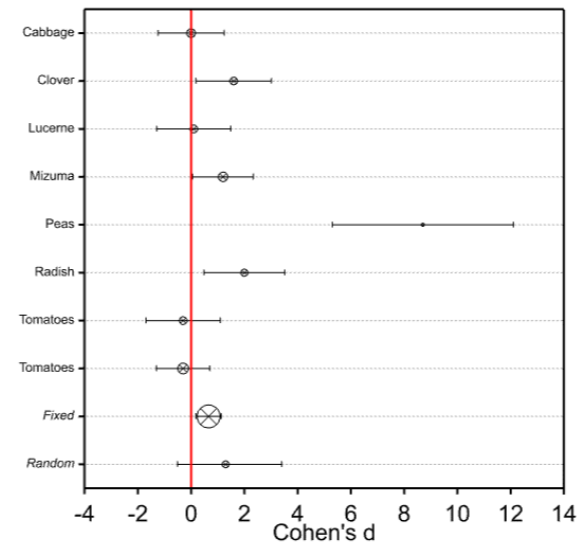

Yates Seaweed

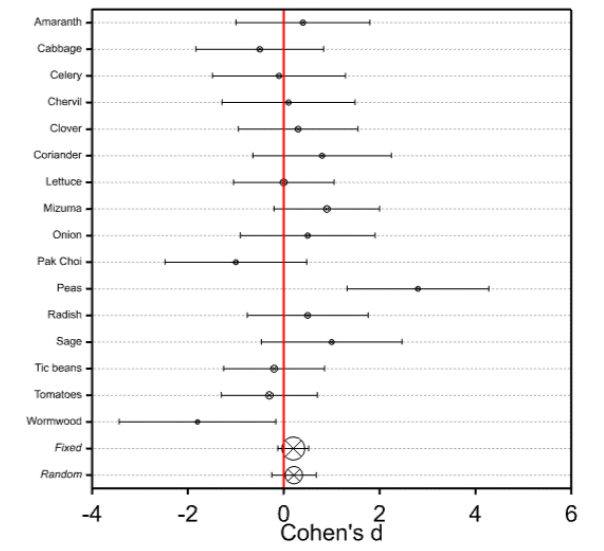

Supplement: Supplementary file 1 [file plants-10-00660-s001.zip › Supplemental File S2 - REML Forest Diagrams.pdf]
